# Biopsychosocial predictors and trajectories of work participation after transdiagnostic occupational rehabilitation of participants with mental and somatic disorders: a cohort study

**DOI:** 10.1186/s12889-018-5803-0

**Published:** 2018-08-15

**Authors:** Karen Walseth Hara, Johan Håkon Bjørngaard, Henrik Børsting Jacobsen, Petter C. Borchgrevink, Roar Johnsen, Tore C. Stiles, Søren Brage, Astrid Woodhouse

**Affiliations:** 1 Department of Public Health and Nursing, Faculty of Medicine and Health Sciences, Norwegian University of Science and Technology (NTNU), Trondheim, Norway; 2 Norwegian Advisory Unit on Complex Symptom Disorders, St. Olavs University Hospital, Trondheim University Hospital, Trondheim, Norway; 3 Department of Circulation and Medical Imaging, Faculty of Medicine and Health Sciences, Norwegian University of Science and Technology (NTNU), Trondheim, Norway; 4 The Norwegian Labour and Welfare Service of Trøndelag, Trondheim, Norway; 5 Forensic Department and Research Centre Brøset, St. Olavs University Hospital, Trondheim University Hospital, Trondheim, Norway; 6 Hysnes Rehabilitation Center, St. Olavs University Hospital, Trondheim University Hospital, Trondheim, Norway; 7 Department of Psychology, Faculty of Social Sciences and Educational Sciences, Norwegian University of Science and Technology (NTNU), Trondheim, Norway; 8 The Norwegian Directorate for Labour and Welfare, Oslo, Norway; 9 Department of Pain Management and Research, Division of Emergencies and Critical Care, Oslo University Hospital, Oslo, Norway

**Keywords:** Mental disorders, Chronic pain, Musculoskeletal diseases, Fatigue, Vocational rehabilitation, Return to work, Unemployment, Prognosis, Comorbidity, Acceptance and commitment therapy

## Abstract

**Background:**

Group-based transdiagnostic occupational rehabilitation programs including participants with mental and somatic disorders have emerged in clinical practice. Knowledge is sparse on subsequent participation in competitive work. This study aimed to investigate trajectories for (re)entry to work for predefined subgroups in a diagnostically heterogeneous sample of sick-listed participants after completing occupational rehabilitation.

**Methods:**

A cohort of 212 participants aged 18–69 on long-term sick leave (> 8 weeks) with chronic pain, chronic fatigue and/or common mental disorders was followed for one year after completing a 3½-week rehabilitation intervention based on Acceptance and Commitment Therapy. Self-reported, clinical and registry data were used to study the associations between predefined biopsychosocial predictors and trajectories for (re)entry to competitive work (≥ 1 day per week on average over 8 weeks). Generalized estimating equations analysis was used to investigate trajectories.

**Results:**

For all biopsychosocial subgroups (re)entry to work increased over time. Baseline employment, partial sick leave and higher expectation of return to work (RTW) predicted higher probability of having (re)entered work at any given time after discharge. The odds of increasing reentry over time (statistical interaction with time) was weaker for the group receiving the benefit work assessment allowance compared with those receiving sickness benefit (OR = 0.92, *p* = 0.048) or for those on partial sick leave compared with full sick leave (OR 0.77, *p* < 0.001), but higher for those who at baseline had reported having a poor economy versus not (OR 1.16, *p* = 0.010) or reduced emotional functioning compared with not (OR 1.11, *p* = 0.012). Health factors did not differentiate substantially between trajectories.

**Conclusions:**

Work participation after completing a transdiagnostic occupational rehabilitation intervention was investigated. Individual and system factors related to work differentiated trajectories for (re)entry to work, while individual health factors did not. Having a mental disorder did not indicate a worse prognosis for (re)entry to work following the intervention. Future trials within occupational rehabilitation are recommended to pivot their focus to work-related factors, and to lesser extent target diagnostic group.

## Background

In real world settings, general practitioners and other health care workers encounter work-disabled populations that are heterogeneous - both in terms of diagnostic variation and varying proximity to the work force. Despite this, most research within occupational rehabilitation has been on relatively homogenous populations (e.g. diagnose-specific rehabilitation [1–3] and programs for the employed [4]. The need for research that traverses diagnostic boundaries has been pointed out [5–7]. This paper adds to the literature by analyzing patterns of work participation following occupational rehabilitation in a study population that is diverse - both diagnostically and with regard to their work affiliation.

Common mental and musculoskeletal disorders are the two major diagnostic categories associated with long-term sick leave [8, 9]. Comorbidity and multiple health complaints are prevalent in the work-disabled population [10–13]. Despite this, national social security databases usually register cause of sick leave as a single diagnostic entity and use this for administrative and policy planning purposes [14]. Selecting one specific “diagnosis of sick leave” can be both clinically challenging and misleading [15]. Persons on sick leave often have combinations of mental and somatic health problems, and a firm division between somatic and psychiatric care is increasingly being questioned [16–18]. Furthermore, diagnostic classification systems, such as ICPC-2 [19] and ICD-10 [20], have shortcomings with regard to compatibility and ability to distinguish between different clinical entities [15]. Diagnostic classification is not consistent among general practitioners assessing patients with subjective health complaints [21]. General practitioners express that it is challenging to use clinical guidelines developed for single diseases when handling patients with multimorbidity [22].

Generic return-to-work (RTW) interventions and intervention components that are effective across diagnoses have been identified [23, 24]. In practice, RTW interventions including participants with mental and/or somatic disorders have been introduced [25–28]. Such transdiagnostic programs are of interest for several reasons: Reduced cost compared to delivering multiple disorder-specific programs, efficient use of rehabilitation personnel, increased access to rehabilitation services and application of therapy that also targets comorbidity [29, 30].

Research within occupational rehabilitation has primarily targeted those attempting to return or stay in the work force. However, interventions are also essential for persons attempting to engage in work for the first time, or after a longer period of unemployment [31, 32]. Government programs are enforced to promote inclusion of the mentally and physically disabled into the work force [9] Mental disorders represent the most frequent and increasing cause of permanent disability, and are particularly rising among young people of working age [33] - a group struggling to start their working lives. In this study, special attention is given to different modes of categorizing persons with mental health problems.

The complex characteristics of a real-world population struggling to either stay in or enter the work force was confirmed in two of our recent papers [34, 35]. The same population is studied in this paper. These were participants on long-term sick leave (> 8 weeks) referred from general practice to an occupational rehabilitation program with an open-door policy regarding employment status and diagnoses. We found that 78% of participants showed overlap of self-reported health problems such as mental distress, chronic pain and chronic fatigue, indicative of high comorbidity. In addition to diagnostic complexity the same population also showed large variation in proximity to the work force. Forty percent were unemployed. All were receiving temporary medical benefits of which 57% were receiving work assessment allowance and the remaining sickness benefit. Work assessment allowance is a benefit indicating work disability of more than 1 year’s duration or a weak connection to the work force. The prevalence of mental conditions in this population varied considerably depending on the route of diagnostic data-collection: by self-report 62%, by sickness certificate of sick leave 38% and by pre-rehabilitation interdisciplinary evaluation including assessment by a psychologist 22%.

Studies on the patterns of (re)entry to competitive work of real world populations are needed to inform clinical, workplace and policy decision-making processes and to guide the utilization of big data in the social security setting. Many studies lack the requisite range of variables to satisfy contemporary biopsychosocial models of return to work and disability [36, 37]. In this paper we will explore associations between a wide range of biopsychosocial characteristics and (re)entry to work in the heterogeneous population described above. We respond to the call for investigations that use a longitudinal RTW outcomes [37, 38] and explore work participation patterns in real-world, heterogeneous populations [36] that show variation in diagnoses [6, 23] and proximity to the work force [31].

### Aims

The aim is to study (re)entry to work after occupational rehabilitation in a sick-listed population that is heterogeneous in terms of diagnoses, length of work disability and employment status. Work participation is investigated as it changes over the course of 56 weeks following completion of a common, on-site occupational rehabilitation program. We compare work trajectories for subgroups of participants that were grouped by biopsychosocial factors. The aim is approached through the following research questions:

1. Is there a difference in the *level of (re)entry to work* (consistent differences in the estimated probabilities of work participation after baseline) between subgroups of expected biopsychosocial predictors of work participation?
2. Is there a difference in the *trend for reentry* to work (odds of average linear reentry over time) between subgroups of expected biopsychosocial predictors of work participation?
3. Are specific patterns of reentry (level and trend) discernable within grouping factors, when various sources of diagnostic data on mental health problems (self-report, sickness certification or interdisciplinary assessment) are used?

## Methods

### Design

This cohort study is nested in a randomized controlled study investigating effects of boosted RTW follow-up by telephone. ClinicalTrials.gov. No. NCT01568970 [35]. Reporting is according to STROBE guidelines [39].

### Participants

Sick listed individuals on temporary work-disability benefits participated in an Acceptance and Commitment Therapy-based occupational rehabilitation program of 3½ weeks duration. Upon admission, they were consecutively invited to participate in the study (January 2012–June 2013). Participants had been referred to the program by general practitioners or other medical specialists and had prior to admission been assessed by an interdisciplinary team consisting of a medical doctor, a psychologist and a physiotherapist.

### Eligibility and exclusion criteria

Participants should be ages 18–59 years old and have a musculoskeletal or other chronic pain disorders, chronic fatigue or a common mental disorder. Further diagnostic criteria were not advised upon referral to allow for inclusion of a real-world population.

Participants should have received sickness certification and be recipients of a temporary medical benefit due to work incapacity (duration over 8 weeks, partial or full-time). By the Norwegian welfare system this involves either *sickness benefit* (compensation for loss of income) or *work assessment allowance* (for those who have either already received sickness benefits for the maximum period of 52 weeks, or have not earned the right to sickness benefits through previous work-related income).

Participants should have a self-defined goal of increasing participation in competitive work, be adequately treated for health problems demanding acute care. To participate in the group-based on-site program participants needed to be able to communicate in Norwegian and maintain basic daily care for themselves during a stay at the rehabilitation center.

Exclusion criteria were severe mental illness (ongoing mania, psychosis or suicidal ideation), active substance abuse and addiction, pregnancy, planning to enter/return to studies rather than competitive work, incomplete study registration procedure or not completing the rehabilitation program due to acute injury/disease or family reasons.

### Study setting

Internet-based self-report questionnaires were filled out by participants prior to entering the rehabilitation program. Pre-admission assessment was at an outpatient department of the university hospital while participation in the onsite 3-½ week program took place at an affiliated rehabilitation center. The rehabilitation program was group-based but included individual sessions. Acceptance and Commitment Therapy was an integrated part of mental training, physical training and work-related problem solving. Collaboration with community stakeholders (the general practitioner, the work place, the social security office and others) was initiated on-site and participants prepared a personal action plan for increasing work participation. The on-site program is further described elsewhere [40].

### Data collection

Baseline self-reported data, clinician reported data and registry-based social security data were collected prior to entering the program. The Norwegian Labour and Welfare Service provided data on employment state, working hours and benefits; both medical (sickness benefits, work assessment allowance and permanent disability benefits) and non-medical (unemployment benefits, parenting benefits and social benefits). The procedure for data collection is described in detail elsewhere [35].

### Outcome (dependent variable)

Main outcome was (re)entry to the ordinary work force analyzed from baseline and up to 56 weeks after completing the rehabilitation program. The outcome variable was dichotomous and defined as *participation in competitive work ≥ 1 day (7.5 h) per week on average over 8 weeks*. This is considered to be substantial enough to represent a meaningful “first step” towards entering the ordinary work force and a relevant cut off for individuals with an anticipated poor prognosis for work. For sensitivity analyses we used the outcome measure *“minimum half time work”* defined as ≥2.5 days (18.75 h) per week on average over 8 weeks. This outcome explores work participation among participants with a stronger connection to the work force. *Levels of reentry to work,* defined as differences in the estimated probabilities of work participation at all time points after baseline and *trend of (re)entry to work,* defined as the odds of average reentry over time, from baseline to end of follow-up were analyzed for all grouping factors.

### Baseline predictors (independent variables)

Variables used as grouping factors were selected a priori among expected predictors of RTW [7, 36] and variables of particular interest to the project, for example therapy-related factors thought to be modifiable ‘. psychological flexibility [41]), benefit-related factors (e.g. grading of sick leave) expected to influence work participation [42] and health-related factors (e.g. diagnosis) used in clinical decision support and to inform policy planning in mental health care and work disability management. Predictors of (re)entry to work spanned major biopsychosocial categories and included individual (micro level) and system (meso and macro level) factors [6]. Variables were divided into five main categories:

1. *Sociodemography* (individual factors): gender, age, level of education and personal economy.
2. *Work and benefits* (system factors): employment state, type of benefit, grading of sick leave and type of work.
3. *Psychological* (individual factors, both general and work-related): expectations of return to work, psychological flexibility and work self-efficacy.
4. *Health* (individual factors): participants’ self-reported health-measures for chronic pain, chronic fatigue, mental distress and sleep disturbance and clinical diagnoses from two different sources (sickness certification by the general practitioner and pre-rehabilitation assessment by the interdisciplinary team)
5. *Functioning* (individual factors): role limitations due to physical problems and role limitations due to emotional problems.

Variables were dichotomized into clinically recognizable sub-groups to support easy use and meaningful interpretation in the health and social service setting.

### Sociodemography

*Gender, age, level of education* and *personal economy* were self-reported. Age was dichotomized at *≥36 years old* since this is a legislative cut-off used in the Norwegian social security system when applying for special disability benefits for young people [43]. The participant’s financial situation was classified using the single item “How do you assess your economy?” The response alternatives are “good, medium, poor”. Participants answering “poor” were registered as having a poor economy.

### Work and benefits

Registry data on *employment state (*employed/unemployed)*, type of benefit* (sickness benefit/ work assessment allowance) and *grading of sick leave* (out of work and on a full benefit/ part-time work combined with a partial benefit) was used. *Type of work* was classified as physically demanding job (no/yes) based on self-report from The Psychosocial Assessment Instrument (PAI) using one single item: “Is your work very physically demanding?”. The response alternatives are “not at all, a little, quite a bit, much”. A cut-off was set at ≥ “quite a bit”. PAI was developed by two experienced occupational psychologists to address psychosocial factors that could predict the course of somatic symptom disorders.

### Psychological

*Psychological flexibility* was assessed using the 7-item (short version) of the Acceptance and Action Questionnaire (AAQ-II) [44]. Each item has seven responses ranging from 1(never true) to 7(always true). Higher scores indicate psychological inflexibility and experiential avoidance*.* Items on the AAQ-II correspond to the processes detailed by the ACT model (action, self-as-context, presence in the moment, values, cognitive defusion and acceptance). The AAQ-II has shown satisfactory structure, reliability and validity [44, 45]. The cut-off was set at > 28, the upper limit of the range considered to indicate a clinically relevant level of distress [44]. Psychological flexibility was of particular interest as a predictor of work since it is amenable to change [36] and specifically targeted through acceptance and commitment therapy [46], the method used in the rehabilitation program.

*Expectation of RTW* was measured through a single item of the Fear Avoidance Beliefs Questionnaire (FABQ) – Work Subscale [47]. The item used asked “I do not think that I will be back in my ordinary work within three months” with 7 item responses range 0 to 6, with a higher score indicating a worse fear of not returning to work [47]. The cut-off was set at ≥3 indicating a non-positive expectation defined as an “uncertain or even poorer expectation of RTW”.

*Work self-efficacy* was tapped using an item made for this study: “How strong is your belief that you will cope with functioning in the ordinary work force” and had eleven numeric response categories from 0 to 10, with higher scores indicating higher self-efficacy. The cut- off was set at > 5.

### Health-related factors

#### Interdisciplinary diagnosis

Clinical assessment for current mental disorder was done by a psychologist as part of the interdisciplinary assessment prior to admission. Self-reported results from the Psychiatric Diagnostic Screening Questionnaire (PDSQ) [48] were followed up clinically with the Structured Clinical Interview for DSM-IV Axis 1 disorders (SCID-I) [49]. The PDSQ is a validated 126-item screening tool that screens for the 13 most common “Diagnostic and Statistical Manual of Mental Disorders, Version IV” (DSM IV) disorders among individuals 18 years of age and older (major depressive disorder, generalized anxiety disorder, panic disorder, posttraumatic stress disorder, alcohol abuse/dependence, drug abuse/dependence, psychosis, bulimia/binge-eating disorder, somatization disorder, obsessive-compulsive disorder, social phobia, hypochondriasis, agoraphobia). It has been shown to have a high sensitivity, but somewhat lower specificity [48]. If the participants scored above the cut-off on the PDSQ, or if the psychologists found other indications of psychiatric disorders, the participants were interviewed according to the SCID-I. Participants were registered as having a mental disorder if the presence of a disorder was confirmed by the SCID-I interview.

#### Main diagnose of sickness certification

Diagnostic data from sickness certificates were retrieved from the Norwegian Labour and Welfare Service and used to categorize the main health-related cause of sick leave. This diagnosis had been set pre-rehabilitation by the treating doctor, usually the general practitioner, using the international classification of primary care (ICPC-2) [14].

The variables used to define self-reported chronic pain, chronic fatigue, mental distress and sleep disturbance are briefly described below and more extensively described elsewhere [35].

#### Self-reported chronic pain

was measured using a single item from the Short Form 8*: “How much bodily pain have you had the last week?” [50]. This item has been validated and used as a proxy measure of chronic pain in Norwegian population studies, using a cut-off at moderate pain or more [51]. Chronic pain was defined if the clinical reports confirmed duration of 6 months or more [51].

#### Self-reported chronic fatigue

was measured using the 13-item Chalder Fatigue Scale [52]. The cut-off was set at a score of ≥4 combined with symptom duration of 6 months or more. This is the recommended cut-off when using the 13-item Chalder Fatigue Scale that has been validated for a Norwegian population [53].

#### Self-reported mental distress

The 14-item Hospital Anxiety and Depression Scale (HADS) [54] measures mental distress, and is divided in an anxiety and a depression scale, each with 7 items. Twenty-one is the maximum score on each scale. The cut-off for mental distress was set at a score ≥ 8 on either the anxiety and/or the depressive scales [55] and is validated for a Norwegian population [56].

#### Self-reported sleep disturbance

The 7-item Insomnia Severity Index (ISI) measures the nature, severity and impact of insomnia symptoms the last 2 weeks and is considered a good outcome measure for insomnia in clinical trials [57]. Reliability and validity are good [58]. The recommended cut-off to identify patients with clinically significant insomnia is ISI ≥ 11 [59].

### Functioning

The Short-Form 8 measures quality of life through eight subscales with one item per subscale [50]. Two of the single item subscales assess role limitations: *The Role-Physical (RP)* item assesses role limitations due to physical health problems with responses ranging from “Cannot perform daily work at or away from home as a result of physical health” to “No difficulty performing daily work at or away from home as a result of physical health”. *The Role-Emotional (RE)* item assesses role limitations owing to personal or emotional problems with response ranging from “Cannot perform work, school, or other daily activities due to personal or emotional problems” to “No limitations in performing work, school, or other daily activities due to personal or emotional problems”. Both items capture the impact of role limitations on performing work or other usual activities. Each item has 5 response categories from 1 to 5. The cut-off was set at ≥3 indicating role limitations ranging from a bit to total limitation.

### Statistical methods

Descriptive data are provided to describe the population at baseline. Regression analysis for repeated measures is used to study trajectories for work participation. Specifically, for each biopsychosocial variable we used generalized estimated equations (GEE) regression analysis to analyze the dichotomous outcome variable (≥ 1 day of competitive work per week) using repeated measurements, an unstructured working correlation structure and treating time as a categorical variable. Each 8-week follow-up period was added to the model as a dummy variable (i.e. weeks 1–8, weeks 9–16, weeks 17–24, weeks 25–32, weeks 33–40, weeks 41–48, weeks 49–56) and the first 8-week period prior to and including time of entry to the program was used as reference category. Precision was measured with 95% confidence intervals (CI). To assess differences between subgroups during follow-up, interaction terms between the studied variable and each registration time-point were included in the model. Estimated marginal values are presented as graphs showing trajectories of work participation.

Distinct levels of (re)entry to work over time within subgroups were assessed for substantial difference in probability for work participation within a subgroup (e.g. employed compared to unemployed) for all time periods after rehabilitation. Substantial differences were indicated by the combination of consistent differences in the estimated probabilities of (re)entry after baseline together with adequate precision of the estimated probabilities at the level of 95% confidence.

We also tested for time-trend differences between subgroups of the investigated variables with time as a continuous variable, i.e. statistical interaction between groups of interest and time (per 8 week periods). Odds ratios (OR) are reported. All models were adjusted for age, gender and the underlying intervention of the randomized controlled trial. To support generalizability of results across non-standardized populations and to allow for comparison of trajectories across correlated diagnostic mental health categories the grouping factors were analyzed separately rather than entered into a more conventional multivariable model. Sensitivity analysis is performed for levels of (re)entry using the outcome measure *“minimum half time work”* (≥2.5 days (18.75 h) per week on average over 8 weeks) and otherwise identical analysis. Data analysis was performed using STATA version 14.2 (StataCorp. 2015. College Station, Texas, USA).

## Results

### Participants

All 278 participants admitted to the on-site rehabilitation program were assessed for eligibility. Of these 29 patients were not eligible, 36 patients declined to participate and one participant was lost to follow-up. This left 212 participants to enter the analysis. The flow of participants is reported elsewhere [35].

### Baseline characteristics

Baseline characteristics are reported in detail in Table 1.

**Table 1:** Baseline characteristics of the study population

Biopsychosocial factors	n/N	Percent
Sociodemography		
Female	169/212	80
Age ≥ 36 years old	154/212	73
Higher education	88/212	42
Poor economy	43/212	20
Work and benefits		
Employed	127/212	60
Work assessment allowance (type of benefit)	120/212	57
Partial sick leave	76/212	36
Physically demanding job	99/190	52
Psychological (attitudes, beliefs or behaviors)		
Low expectation of return to work	67/189	35
High psychological flexibility	44/193	23
High work self-efficacy	129/163	79
Health		
Chronic pain by self-report	159/211	75
Chronic fatigue by self-report	166/211	79
Mental distress by self- report	131/211	62
Mental disorder as main diagnosis on sickness certificate	73/197	37
Mental disorder confirmed by interdisciplinary assessment	45/211	21
Sleep disturbance by self-report	76/212	36
Functioning		
Role limitations because of emotional problems	124/210	59
Role limitations because of physical health	144/212	68

#### Sociodemography

Average age of participants upon entry to the program was 42 years (range 20–59). Among participants 80% were women, 42% had higher education (completed college/university education) and 20% reported having a poor economy.

#### Work and benefits

All participants were on temporary medical benefits; 43% received sickness benefits and 57% work assessment allowance. In total 60% were registered as employed prior to admission. 15% of participants self-reported that it was more than 3 years since they had been in work (*n* = 28) or that they had never been in work (*n* = 3). 36% of the participants were on partial sick leave (a combination of part time paid work and receiving a partial temporary medical benefit), while the remaining 64% were on full-time sick leave. Fifty-two percent reported having had a physically demanding job.

#### Psychological factors

Among participants 23% showed high psychological flexibility, 35% low expectation of returning to work and 79% high work self-efficacy.

#### Health (symptoms and diagnoses)

Participants self-reported clinically significant symptom levels as follows: 75% chronic pain (of at least moderate intensity), 79% chronic fatigue, and 62% mental distress. Seventy-eight percent of participants presented with overlap of these conditions, indicating comorbidity.

The main medical causes of sick leave as specified on the medical certificates using International Classification of Primary Care (ICPC-2) were: 37% mental disorder, 30% musculoskeletal disease, 20% general/unspecified disease, and 7% neurological disease. The remaining 5% belonged to a broad diagnostic category including digestive, respiratory, dermatological, reproductive and endocrine diseases.

Prior to entering the program participants completed interdisciplinary assessment including consultation with a psychologist. By this, 21% of participants were diagnosed with a mental disorder according to DSM-IV using SCID-I. This was further translated into an ICD-10 diagnosis. The following mental disorders were diagnosed: Mild to moderate depressive disorder (*n* = 24), anxiety disorder (*n* = 17), post traumatic stress disorder (*n* = 1), adjustment disorders (*n* = 4) somatoform disorder (*n* = 3), eating disorder (*n* = 2), substance abuse (*n* = 1). Seven participants had more than one mental disorder.

#### Functioning

68% of participants reported role limitations because of physical health and 59% due to emotional problems.

### Outcome

**Fig. 1:**
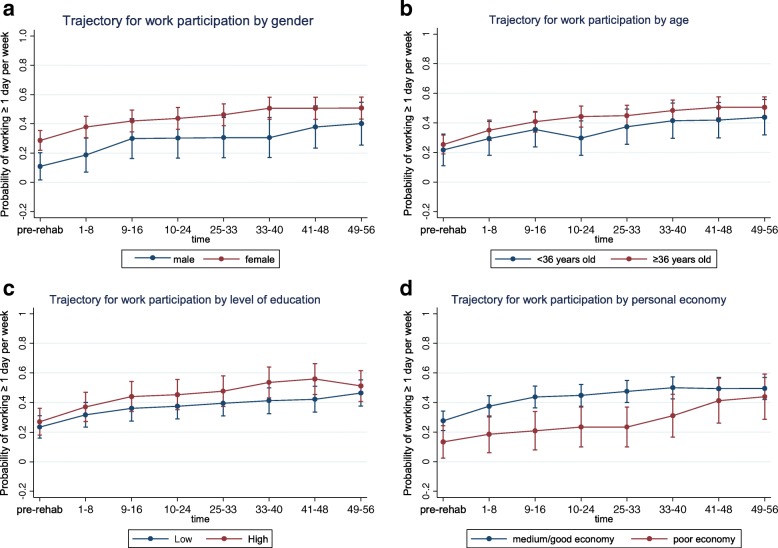
Sociodemography: Generalized estimating equations analysis (GEE) of work participation over the first year after completing on-site rehabilitation. Participants are subgrouped by sociodemographic factors and their associated trajectories of work participation are presented. **a** Trajectory for work participation by gender. **b** Trajectory for work participation by age. **c** Trajectory for work participation by level of education. **d** Trajectory for work participation by personal economy

**Fig. 2:**
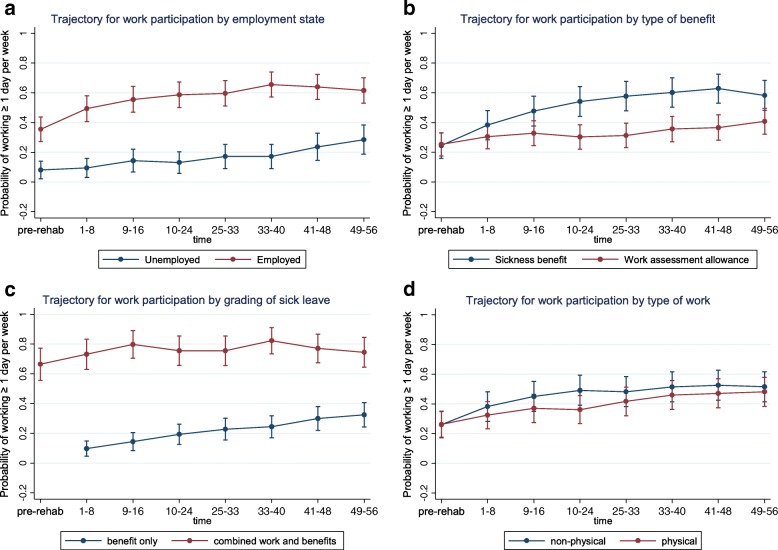
Work and benefit factors: Generalized estimating equations analysis (GEE) of work participation over the first year after completing on-site rehabilitation. Participants are subgrouped by work and benefit factors and their associated trajectories of work participation are presented. **a** Trajectory for work participation by employment state. **b** Trajectory for work participation by type of benefit. **c** Trajectory for work participation by grading of sick leave. **d** Trajectory for work participation by type of work

**Fig. 3:**
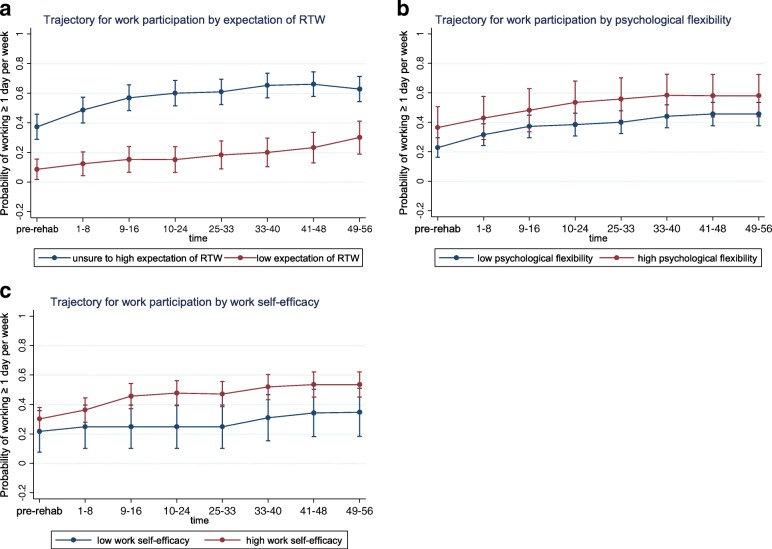
Psychological factors: Generalized estimating equations analysis (GEE) of work participation over the first year after completing on-site rehabilitation. Participants are subgrouped by psychological factors and their associated trajectories of work participation are presented. **a** Trajectory for work participation by expectation of RTW. **b** Trajectory for work participation by psychological flexibility. **c** Trajectory for work participation by work self-efficacy

**Fig. 4:**
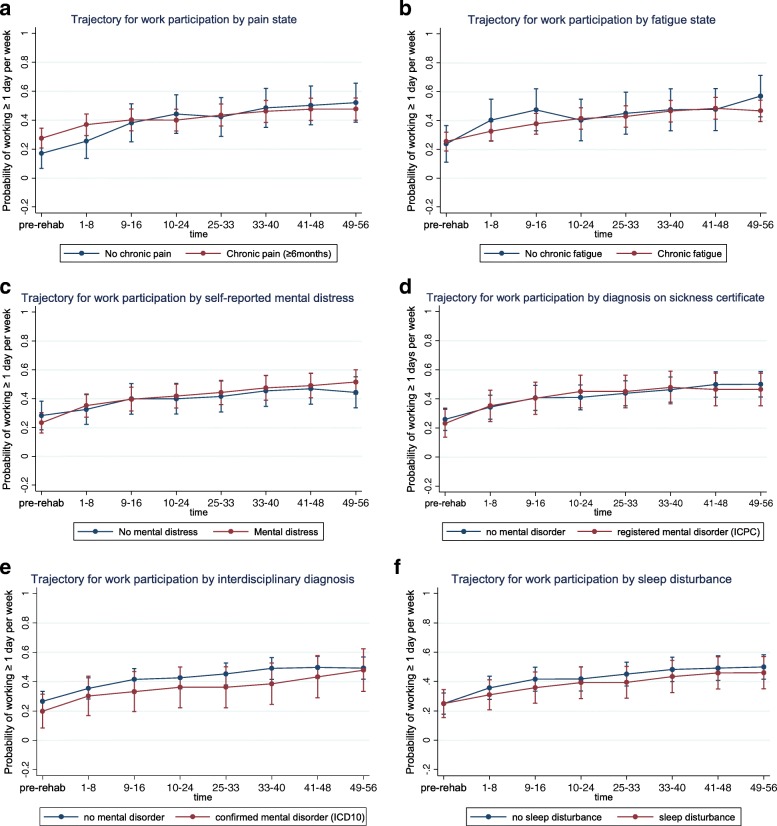
Health-related factors: Generalized estimating equations analysis (GEE) of work participation over the first year after completing on-site rehabilitation. Participants are subgrouped by health-related factors and their associated trajectories of work participation are presented. **a** Trajectory for work participation by pain state. **b** Trajectory for work participation by fatigue state. **c** Trajectory for work participation by self-reported mental distress. **d** Trajectory for work participation by diagnosis on sickness certificate. **e** Trajectory for work participation by interdisciplinary diagnosis. **f** Trajectory for work participation by sleep disturbance

**Fig. 5:**
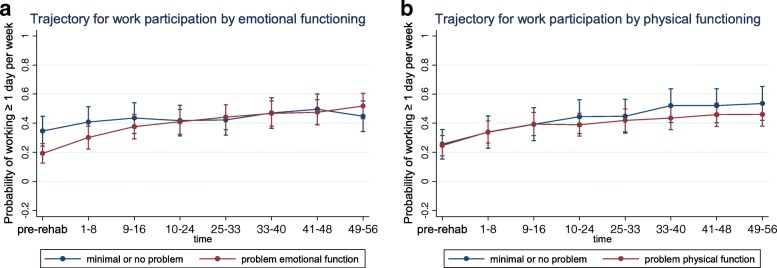
Functioning: Generalized estimating equations analysis (GEE) of work participation over the first year after completing on-site rehabilitation. Participants are subgrouped by functioning factors and their associated trajectories of work participation are presented. **a** Trajectory for work participation by emotional functioning. **b** Trajectory for work participation by physical functioning

Trajectories for (re)entering the work force during the first year after the rehabilitation program are presented in (Figs. 1, 2, 3, 4 and 5) with separate graphs for each grouping factor. Each figure represents one of the five categories of biopsychosocial variables.

#### Levels of (re)entry

Distinct *levels of work (re)entry,* defined as a substantial differences in estimated probabilities of reentry persisting throughout the first year post-discharge were identified for several factors. This is visualized in Figs. 1, 2, 3, 4 and 5 as subgroups with non-overlapping confidence intervals at all time-points after baseline. Findings for differences in levels of entry are summarized in Table 2.

**Table 2:** Overview of differences in levels of (re)entry^a^ to work, i.e. estimated probability of work participation measured at all time points after participation in the rehabilitation program

Higher level of (re)entry^a^ for the following subgroups:	Lower level of (re)entry^a^ for the following subgroups:
Substantially^b^ higher probability of work participation after baseline:	Substantially^b^ lower probability of work participation after baseline:
Employed (compared to unemployed)	Low expectation of return to work (compared to high or unsure)
Partial sick leave (compared to full sick leave)	
Higher probability of work participation after baseline, but not a substantial difference:	Lower probability of work participation after baseline, but not a substantial difference:
Female (compared to male)	Poor economy (compared to medium/good)
Age ≥ 36 years old (compared to younger)	Work assessment allowance (compared to sickness benefit)
Higher education (compared to lower)	Physically demanding job (compared to not physically demanding)
High psychological flexibility (compared to low)	Sleep disturbance (compared to no sleep disturbance)
High work self-efficacy (compared to low)	

Grouping factors with no consistent differences in levels of (re)entry^a^ after participation in the program:

Chronic pain, fatigue, mental distress, mental disorder as main diagnosis on sickness certificate, mental disorder confirmed by interdisciplinary assessment, role limitation due to emotional problems or role limitation due to physical health

^a^(Re)entry to work is defined as participating in paid work ≥1 day (7.5 h) per week on average for 8-week periods. Participants were subgrouped according to baseline characteristics

^b^A substantial difference in level of (re)entry is indicated by the combination of a consistent difference in the estimated probabilities of (re)entry after baseline and a comparatively high precision of the estimated probabilities at the level of 95% confidence

Substantially *higher levels* of (re)entry are observed throughout the first year for participants that were registered as employed upon entry to the program (Fig. 2a) or were on partial sick leave (Fig. 2c). Although otherwise lacking precision (seen in graphs as overlapping confidence intervals) estimates similarly showed higher probabilities of participation throughout the first year for participants who were either female (Fig. 1a), of higher age, i.e. ≥ 36 years old (Fig. 1b) had a higher education (Fig. 1c), had higher psychological flexibility (Fig. 3b) or higher work self-efficacy (Fig. 3c).

Substantially *lower levels* of (re)entry throughout the first year were seen for participants with low expectation of return to work (Fig. 3a). Although otherwise lacking precision, estimates similarly showed lower probabilities of participation throughout the first year for participants with a poor economy (Fig. 1d), those receiving work assessment allowance (Fig. 2b), a physically demanding job (2d) or a sleep disturbance (4f).

Health factors such as self-reported chronic pain (Fig. 4a), self-reported chronic fatigue (Fig. 4b), self-reported mental distress (Fig. 4c), mental disorder as main diagnosis on sickness certificate (Fig. 4d), and mental disorder as interdisciplinary diagnosis (Fig. 4e) did not discriminate between levels of participation.

Self-reported role limitations at baseline due to emotional problems (Fig. 5a) or physical health (Fig. 5d) did not discriminate between levels of participation.

#### Trends of (re)entry

Different linear trends of (re)entry were identified if the odds of average reentry over time was substantially higher for one of the two categories within the grouping factor. Analysis for statistical interaction with time showed that participants who at baseline had either reported a poor economy (OR 1.16, *p* = 0.010, CI 1.030–1.299) or problems with participation due to emotional functioning (OR 1.11, *p* = 0.012, CI 1.023–1.200) showed a stronger trend for (re)entering work when compared to their counterpart subgroups (Table 3). On the contrary, participants who at baseline were receiving work assessment allowance (OR = 0.92, *p* = 0.048, CI 0.849–0.999) or on partial sick leave (OR 0.77, *p* < 0.001, CI 0.684–0.857) showed a weaker trend for (re)entering work when compared to their counterpart subgroups (Table 3). None of the subgroups for health-related or psychological factors substantially differentiated trends for RTW.

**Table 3:** Subgroups of expected biopsychosocial predictors are compared for linear trend of average (re)entry to work per 8-week period. (Re)entry is defined as “participating in paid work ≥ 1 day (7.5 hours) per week on average for 8 week periods” and is measured throughout 56 weeks after on site rehabilitation. The reported results are from generalized estimating equations (GEE) analysis of the additional effect of time within subgroups (statistical interaction with time)

Biopsychosocial factors	Odds ratio	95% Confidence intervals	
		Lower	Upper
Sociodemographic			
Female (compared to male)	0.97	0.871	1.086
Age ≥ 36 years old (compared to younger)	1.00	0.908	1.096
Higher education (compared to lower)	1.01	0.935	1.098
*Poor economy (compared to medium/good))*	*1.16*	*1.030*	*1.299*
Work and benefits			
Employed (compared to unemployed)	0.92	0.821	1.021
*Work assessment allowance (compared to sickness benefit)*	*0.92*	*0.849*	*0.999*
*Graded sick leave (compared to full sick leave)*	*0.77*	*0.684*	*0.857*
Physically demanding job (compared to not physically demanding job)	1.03	0.950	1.127
Psychological			
High psychological flexibility (compared to low)	0.99	0.896	1.093
Low expectation of return to work (compared to high or unsure)	1.08	0.968	1.212
High work self-efficacy (compared to low)	0.98	0.883	1.091
Health			
Chronic pain (compared to no chronic pain)	0.95	0.861	1.042
Chronic fatigue (compared to no chronic fatigue)	0.98	0.893	1.086
Mental distress (compared to no mental distress)	1.05	0.971	1.145
Mental disorder as diagnosis on sickness certificate (compared to no mental disorder)	0.97	0.891	1.051
Mental disorder by interdisciplinary assessment (compared to no mental disorder)	1.03	0.933	1.137
Sleep disturbance (compared to no sleep disturbance)	0.99	0.915	1.081
Functioning			
Role limitations because of physical health (versus no limitations)	0.95	0.876	1.038
*Role limitations because of emotional problems (*versus *no limitations)*	*1.11*	*1.023*	*1.200*

#### Sensitivity analysis (GEE) for levels of (re)entry

To “minimum half-time work”, that is working at least 2.5 days (18.75 h) per week on average over 8 week periods showed similar patterns as the main analysis with the exception that one additional factor – type of benefit - now showed substantial differences in levels of reentry to work. Participants on work assessment allowance consistently showed substantially lower levels of (re)entry at this higher cut-off when compared to those receiving sickness benefit.

## Discussion

This study gives new insight into the associations between nineteen biopsychosocial factors and trajectories for reentry to competitive work in a long-term sick-listed population that was heterogeneous both in terms of diagnoses (mental and somatic disorders) and affiliation to working life (employed and unemployed, partial and full sick leave, varying duration of benefits). All participants had completed the same occupational rehabilitation intervention based on Acceptance and Commitment Therapy in transdiagnostic groups. Work participation was registered for 56 weeks following the intervention. Results showed distinct (re)entry patterns for several factors: employment state, expectation of work, type and grading of benefits, economic situation and role limitations due to emotional problems. Largely, these factors are related to work and social security benefits. Patterns of (re)entry to work were not substantially different for health factors. During the first year after transdiagnostic rehabilitation, persons with mental health problems showed similar patterns of reentry as those who did not have such problems. The heterogeneous nature of the study population made it possible to compare patterns of work participation among subgroups that are often not included in the same rehabilitation intervention.

### Levels and trends of (re)entry to work (aims 1 and 2)

Participants showed increasing (re)entry to work over time for all of the explored factors, both across and within grouping factors (Fig. 1–5). Lower levels of reentry to work throughout the year correlated with expected negative predictors (e.g. low expectation of return to work) and higher levels of (re)entry correlated with expected positive predictors (e.g. employment and partial sick leave). This same pattern was seen for low precision estimates, except that for health factors consistent level differences were not discernable, perhaps with the exception of mental disorders diagnosed at the level of specialist care. These findings are in accordance with the main body of literature on models and determinants of RTW [7, 60] indicating that expectations of return to work and other work- and system-related factors have greater influence on the process of (re)entry to work than health-related factors. Notably, health factors relating to symptom and diagnosis did not distinguish between those who return to work and those who do not (similar levels and trends of reentry).

For the majority of factors the *trend for (re)entry* was not significantly different between subgroups. Even for subgroups that showed substantially lower levels of (re)entry, e.g. *the unemployed* or those with *low expectancy of RTW*, there is a similar or stronger positive trend (upward pointing slope) of (re)entry throughout the one-year follow-up period. Thus, relative to their poorer starting point these groups in fact showed satisfactory improvement with regard to (re)entry. This is an important distinction and addition to the RTW literature. In clinical practice, negative prognostic factors have commonly been used to disqualify participants from entry to RTW programs, claiming that participants with an assumed poorer prognosis are less likely to draw benefit from programs. Our findings are to the contrary, suggesting that the subgroups of individuals with a weaker starting point (probability of work participation) likewise progressed towards work following transdiagnostic occupational rehabilitation when compared to their counterpart subgroups. The exception was persons on work assessment allowance who did not progress towards work as quickly as their counterparts on sickness benefit.

Work assessment allowance is by legislation a benefit granted to sick listed persons with a weaker connection to the work force, either in terms of lacking or limited previous employment or by having exhausted their right to sickness benefits through sick leave of over 1 years duration. We found a poorer prognosis for (re)entry to work among participants receiving work assessment allowance, seen as both lower level (not substantial) and significantly weaker trend of (re)entry. Sensitivity analysis confirmed that reentry above the “halftime” work (at least 2,5 days per week on average) threshold was substantially lower for those on work assessment allowance than for those on sickness benefit. National reports show high permanent disability and retarded inclusion into the work force for persons on this benefit [61, 62]. Our findings confirm that persons on work assessment allowance, i.e. those with a poor connection to the work force and/or benefits of longest duration, appear to struggle most with (re)entry to work.

Partial sick leave (combination of benefits and paid work) at baseline indicated higher levels of work participation overall, but a poorer trend for increasing entry to work over time, when compared to those on full sick leave. Partial sick leave at baseline seems to protect a relatively stable proportion of the population against falling out of work (at group level), but is associated with an overall slower trend of increasing work participation within the first year. Although not directly comparable, this resonates with previous findings indicating that graded participation supports higher and earlier work inclusion, but may prolong total time on benefits for the group as a whole [63].

### Mental health and (re)entry to work (aim 3)

Similar trajectories for work participation were seen among participants with *mental and non-mental disorders*, regardless of source of diagnostic data (self-reported, by sickness certification or by interdisciplinary assessment). I t is well documented that in the general population persons with mental disorders face higher barriers related to work participation and reentry [9, 64, 65]. Surprisingly, the results suggest that for long-term sick leave and after participating in transdiagnostic rehabilitation, pathways for reentry to work are comparable across common mental and somatic disorders. Results should be interpreted with attention to the fact that we have not studied the average population on sick leave, but rather a sample of persons on long-term sick leave that were referred to rehabilitation in specialist care. Work disability was both long lasting and had not been resolved through primary care and community level rehabilitation efforts. In a recent Norwegian government report it was shown that the hazard rate for return to work fluctuated over time in the same way for sick-listed persons with mental and musculoskeletal disorders [66]. This indicated that other employment related factors factors were more important than health factors. Distinguishing between somatic and mental health problems may be less relevant when long-term work disability is the problem.

Our findings were stable across different sources of diagnostic data, despite the fact that the *prevalence of mental health problems* varied greatly depending on the source of data (21% by interdisciplinary assessment, 38% if sickness certification by a medical doctor and 62% by self report). Researchers, clinicians and policy makers should be aware of the danger of misinterpreting data if single sources are used and/or interpretation is out of context. Multiple data-sources are recommended when discussing mental health problems in the population.

With regard to functioning, participants that experienced role limitations due to emotional problems at baseline had stronger trend for reentry than those not experiencing such. Hypothetically, this could be due to improved emotional functioning following the transdiagnostic intervention, as a result of the intervention and/or the return to work process itself. Future studies may wish to focus on this and other potentially modifiable factors that can be targeted in therapy (e.g. emotional functioning, return to work expectations and psychological flexibility).

### Real-world research and choice of statistical model

The findings from this study fit with an emerging body of evidence [60] suggesting that persons with long term work disability due to common health problems such as common mental disorders and musculoskeletal/pain disorders may best be managed with a holistic and less diagnose-focused approach to occupational rehabilitation. Similarities in etiology, assessment and therapeutic approach that cross diagnostic boundaries have been pointed out [6, 7, 29, 67]. Most studies persist in recruiting participants from relatively homogenous samples and dichotomizing between psyche and soma. Traditional clinical boundaries and the researchers preference of standardized setting to support internal validity are adhered to. However, the problem of generalizability arises, with lack of external validity and subsequent problems with implementation in ordinary practice [68]. Various approaches are recommended when developing complex interventions in real world contexts [69]. This paper adds to the literature on occupational rehabilitation by giving a different perspective on prediction analysis than the preferred multivariable prediction model.

Prediction of outcome for occupational rehabilitation has historically primarily been based on *clinical judgment* of factors that are considered relevant. The range of factors has depended on the applied conceptual model (e.g. biomedical, biopsychosocial, etc.). To increase precision empirical evidence has been applied to create *actuarial models.* The gold standard is the multivariable model, but some weaknesses have been pointed out [70, 71]: Variables are assumed stable and static. Misclassification is risked if there is no room for individual difference. Problems arise if critically important data are not collected or if important variables are imprecisely measured. Many prospective studies are of limited scope and few studies actually cover the requisite range of variables for comprehensive prediction analysis. Over fitting of the model may occur and result in reduced external validity. Generalizability to other populations and contexts is often unknown. Conclusively, actuarial models may not be easily translated into secondary prevention applications. In clinical settings and for policy planning in broad populations, simpler and less conservative prediction models may be more useful and recognizable.

We anticipated that a strict actuarial model might not give reliable results while other possible important associations could be lost [70]. In such situations methodological compromise is recommended. In this study, exploratory analysis using multiple adjusted single factor models was the method of choice. This non-multivariate model is less conservative and has limitations, but was chosen to match the research questions, support generalizability [72] and acknowledge the challenges of complex transdiagnostic interventions in real world research. This simpler model translates for easy use in clinical practice and supports comparison of closely correlated variables of practical interest (e.g. mental disorders diagnosed in different settings). Furthermore, results from single factor prediction models can be used to generate more complex, computer-based prediction models using big data.

### Limitations and strengths

#### Limitations

National setting may influence the results as determinants of RTW may vary due to jurisdictional differences in compensation from employers and social security systems [36].

The results should be interpreted with awareness to the fact that the adjusted single factor model is less conservative than if all factors were studied simultaneously in the same model.

Analysis of known predictors was not exhaustive. Selection was limited by availability of baseline measures. Future studies may wish to focus more broadly on psychosocial work place and other system factors [73].

Single items (e.g. self-efficacy) were constructed for easy use in clinical practice and to encourage higher response rates. Biopsychosocial predictors were dichotomized to support clinically recognizable entities. In pragmatic research it is accepted to construct proxies [74] and single item [75] measures that handle easily in different research and clinical settings as long as this is reported.

#### Strengths

A unique feature of the study is the real world composition of this long term work-disabled and broadly heterogeneous population. This increases external validity and generalizability. Most studies on RTW programs have limited scope and strict inclusion/exclusion criteria.

The investigation of work trajectories is an advantage since return to work is a dynamic process with movement in and out of work. Time to event studies have dominated the field of RTW, but analyzing RTW as a one-time-event can be misleading and does not adequately capture the translational nature of work participation [36, 65, 76]. Longitudinal studies are necessary to identify work patterns over time.

Features that assure high quality of the dataset are the prospective design and the use of multiple data sources (self report, clinical and registry). Missing data was a negligible problem due to near complete registry data combined with high compliance with self-reporting of baseline data, as reported elsewhere [35].

### Implications

This study adds new understanding of the work (re)entry patterns of a complex, real-world population. This knowledge may generate further hypothesis, and be used in developing decision support for general practice and other health and welfare services involved in planning occupational rehabilitation for heterogeneous populations. Prediction rules are typically developed in rather homogenous samples but are practically useful only if they provide accurate risk prediction in different settings [77]. Guidelines for work disability management are recommended further developed with attention to conceptual models that are applicable across health conditions (22, 36).

Our results show that groups that are often viewed as marginalized from the work force (e.g. those with a mental disorder or the unemployed) may progress towards work as much or more after the same transdiagnostic intervention as groups that do not face the same barriers. These marginalized groups showed the same or higher potential for reentry to work (similar or stronger trend) supporting that they should at minimum be offered equal opportunities within work disability management.

Persons at high risk of not being included in the work force may benefit from a transdiagnostic approach that draws attention to work place and social security issues. This is in line with recommendations from other studies within the field of occupational rehabilitation. A related qualitative study on the studied intervention recommended a strengthened focus on specific steps towards return to work [78]. Other RTW interventions have shown that adding workplace strategies may provide enriched work environments that support the underlying therapeutic process [79–81]. This study does not answer how work and social security issues can best be dealt with and future research is needed. Cautionary sub-group analysis from the randomized controlled trial carried out alongside this study indicated that groups with lower probabilities of entering work may benefit from a simple regime of telephone support in the phase of reentering work and daily life [35]. An ongoing randomized controlled trial will study the effect of adding a workplace component to the intervention [82].

This transdiagnostic group-based occupational rehabilitation intervention has shown promising feasibility for implementation and acceptability to participants [26] but needs to be developed further. This is of interest since common rehabilitation programs for mixed groups of participants potentially have financial, practical and therapeutic benefits. Inference cannot be drawn from this study on the effect of the transdiagnostic occupational rehabilitation program, and further research is needed to establish for which persons a transdiagnostic approach to occupational rehabilitation may be preferred, and how transdiagnostic programs should be personalized.

## Conclusion

Interventions in occupational rehabilitation have commonly been designed for relatively homogenous groups of participants. This study adds knowledge on longitudinal associations between biopsychosocial factors and reentry to competitive work in a long-term sick-listed population that was characterized by heterogeneity in terms of diagnoses (mental and somatic disorders) and affiliation to working life (employed or unemployed, partial or full sick leave, varying duration of benefits). Commonly, all participants had completed the same occupational rehabilitation intervention based on Acceptance and Commitment Therapy delivered in transdiagnostic groups. Individual and system factors related to work, such as expectations of RTW, employment status and grading of sick leave could distinguish between pathways for return to work. Health factors were of less importance for work outcomes. The presence of a mental disorder did not indicate a worse prognosis for reentry to work after participation in a transdiagnostic program. Future trials within occupational rehabilitation are recommended to pivot their focus to work-related factors, and to lesser extent target diagnostic group.

## Abbreviations

AAQ II: Acceptance and Action Questionnaire, version II
ACT: Acceptance and Commitment Therapy
CI: Confidence interval
DSM-IV: The Diagnostic and Statistical Manual of Mental Disorders, version IV
FABQ: Fear-Avoidance Beliefs Questionnaire
GEE: General estimating equations
HADS: Hospital Anxiety and Depression Scale
ICD-10: International Classification of Disease, version 10.
ICPC-2: International Classification of Primary Care, version 2
OECD: The Organisation for Economic Co-operation and Development
OR: Odds ratio
PDSQ: Psychiatric Diagnostic Screening Questionnaire
RTW: Return to work
SCID-I: Structured Clinical Interview for DSM-IV Axis I Disorders
